# Mind wandering may both promote and impair learning

**DOI:** 10.3758/s13421-023-01466-8

**Published:** 2023-09-25

**Authors:** Alexander Soemer, Christian Gericke, Ulrich Schiefele

**Affiliations:** 1 Hector Research Institute of Education Sciences and Psychology, University of Tübingen, Europastr. 6, 72072 Tübingen, Germany; 2 Department of Psychology, University of Potsdam, Karl-Liebknecht-Str. 24-25, 14476 Potsdam, Germany

**Keywords:** Mind wandering, Daydreaming, Reading comprehension, Comprehension monitoring, Elaboration

## Abstract

The present investigation deals with individual differences in habitual (trait-level) mind wandering and their effects on learning. We hypothesized that the ‘positive-constructive’ type of habitual mind wandering would promote task-related thinking and the ‘poor-attention’ type to promote task-unrelated thinking. This hypothesis was tested in a study with 200 participants who rated different aspects of their mind wandering in daily life in one session and completed a reading study in a second session. The reading study included thought probes, retrospective questions about readers’ thought contents, and comprehension tests after reading. In line with our hypothesis, data analysis revealed that some forms of positive-constructive mind wandering were positively associated with text-related thought, whereas poor-attention mind wandering was positively associated with text-unrelated thought. The present results add to the literature by emphasizing different types of trait-level mind wandering and their potentially opposite effects on learning.

## Introduction

In recent years, an increasing number of researchers have become interested in a phenomenon called ‘mind wandering’ (MW) that can be defined as a mental state in which an individual’s attention turns to self-generated thoughts resulting in reduced processing of external stimuli (Christoff et al., 2016; Smallwood & Schooler, 2015). The mental state of MW has been of particular interest because of its frequent emergence in situations that require sustained attention to external events and its often negative consequences in many of these situations (Randall et al., 2014, Smallwood & Schooler, 2006, 2015), including learning (e.g., Bonifacci et al., 2022).

One important aspect of MW, however, is that it not only refers to a specific state of an individual’s mind, but also to trait-level differences in the propensity of self-generated thought. Accordingly, people who tend to mind-wander in one situation also tend to do so in others (e.g., Kane et al., 2016; Rummel et al., 2021). Furthermore, individuals differ in a number of phenomenological aspects of their MW episodes such as vividness, emotional valence, and temporal orientation (e.g., Baird et al., 2011; Klinger, 2013; Singer & Antrobus, 1972). However, previous research on individual differences in MW and its consequences for learning has rarely made a distinction between overall MW propensity and different types of habitual MW. Instead, MW has often been equated with poor attention and limited to situations in which a concurrent task requiring attention to external information has to be carried out (Kane & McVay, 2012). This perspective has led to the prevalent view of MW as something that tends to impair any concurrent activity, including learning, and thus should be avoided (but see, e.g., Baird et al., 2012).

In the current article, we take up the idea that individuals differ in the type of MW they habitually engage in and investigate the hypothesis that these differences may exert opposite effects on learning. Specifically, we relate individual differences in ‘positive-constructive’ and ‘poor-attention’ MW, as defined by Huba et al. (1981), to the occurrence of MW episodes in a reading task. Our working hypothesis is that individuals who habitually engage in the poor-attention type of MW experience more text-unrelated thoughts, whereas individuals who habitually engage in positive-constructive MW generate more text-related thoughts that ultimately promote comprehension. Note that the occurrence of MW during specific task situations is termed *task-unrelated thought* (TUT) throughout this article, in accordance with the literature (e.g., Kane & McVay, 2012), whereas an individual’s propensity to engage in MW in various daily-life situations is called *habitual MW*.

### Task-unrelated thought (TUT)

A large part of recent MW research has been concerned with the occurrence of self-generated thoughts that do not have any obvious relation to a concurrent activity (Smallwood & Schooler, 2006, 2015). In a typical study on this topic, participants are periodically interrupted while they are dealing with a task requiring sustained attention on external stimuli such as reading a text (e.g., Soemer & Schiefele, 2019). During these interruptions, participants are asked to indicate whether they were focusing on the task or instead thinking about something task-unrelated immediately before the interruption. All responses that indicate participants were ‘off-task’ are considered as TUTs, and an estimate of TUT rate is then derived by taking the number of off-task responses and dividing them by the number of interruptions.

Researchers have used variations of this paradigm to determine the frequency and the characteristics of TUTs as well as their effects on the concurrent task. It has been found that TUTs occur about 30% of the time in such situations (e.g., Kane et al., 2007, 2017), and that they usually impair performance in many types of tasks, including reading (e.g., McVay & Kane, 2012; Soemer & Schiefele, 2019, 2020), learning foreign vocabulary (Xu & Metcalfe, 2016), or learning from (online) lectures (Szpunar et al., 2013; Hollis & Was, 2016). Moreover, the likelihood of TUTs depend on both task-related and person-related factors. For example, TUT rates appear to be sensitive to difficulty manipulations (Feng et al., 2013; Soemer & Schiefele, 2019), and they depend on individuals’ executive control capabilities (Kane & McVay, 2012; Soemer & Schiefele, 2019, 2020) and motivation-related factors such as interest or motivation (e.g., Seli et al., 2015; Soemer et al., 2023).

Whereas most theorizing about MW (see Schooler et al., 2011; Smallwood & Schooler, 2015; Thomson et al., 2015) has been based on laboratory studies, there are a few studies that were conducted in the field as well. Most prominently, Kane et al. (2007; see also Kane et al. 2017, for a replication) probed their participants in their daily life at various points in time and asked to rate, among other things, their state of mind and aspects of their current activity such as its difficulty. Not only did the participants experience TUTs at similar rates compared to the laboratory (i.e., around 30%), TUT rates correlated well with participants’ executive control capabilities (measured in a separate laboratory session), when their concurrent activities were rated as highly difficult.

Taken together, the available evidence suggests that: (1) TUTs are a frequent phenomenon in both the laboratory and daily life, (2) they usually impair a cognitively demanding concurrent activity including learning activities, and (3) the frequency of TUTs depends on both task context and individual-difference factors.

### Habitual mind wandering (MW)

Although the restriction of MW to TUTs has been successful at explaining many data patterns related to performance impairments in demanding task contexts, TUTs likely only capture a very specific aspect of MW – the ability to focus one’s attention on something that is currently going on in the immediate environment whilst resisting internal sources of distraction (e.g., Kane & McVay, 2012). However, this restriction likely fails to capture forms of self-generated thought that occur in other contexts, yet qualify as MW in the sense that they are independent from the external environment and thematically unconstrained (Christoff et al., 2016). Specifically, this may include thoughts that occur in idle situations (when there is nothing to process), thoughts that occur while someone is engaging in a well-trained activity (e.g., when brushing one’s teeth), or thoughts that occur when self-generated thoughts are potentially beneficial or even necessary, for example, for pursuing future goals (Kvavilashvili & Rummel, 2020) or solving problems (Gericke et al., 2022). The question arises how similar or dissimilar such MW is compared to TUTs – in particular, with regard to its frequency, content, and effect on performance.

Interestingly, there is an older line of research dating back to the 1960s on a related construct named ‘daydreaming’ that has been characterized as a person’s frequency and style of self-generated thought in various life situations (Singer, 1975, 1993). In contrast to more recent investigations of MW as TUTs, daydreaming research considered not only episodes of MW that emerge during the execution of attention-requiring activities, but also in situations lacking strong demands on attention. Furthermore, researchers in the daydreaming tradition have mostly used questionnaires to investigate individual differences in the overall frequency, content, and emotional valence of MW in daily life without requiring participants to carry out a task and without probing or asking them in specific situations or after the execution of a specific task (Singer & Antrobus, 1972).

Apparently, the daydreaming construct largely overlaps with what has been termed MW in more recent times (Stawarczyk, 2018). That is, daydreaming explicitly includes instances of TUT, although these kinds of MW may be subsumed under the umbrella term ‘poor-attention’ daydreaming (Huba et al., 1981). In the following, we use the term ‘habitual MW’ instead of the term ‘daydreaming’ to emphasize this overlap.

There are several important outcomes of daydreaming research that may be transferred to the study of individual differences in habitual MW and their relation to thought processes occurring during learning. First, most human beings seem to habitually engage in MW a substantial amount of time in their daily life and at similar rates as in the laboratory (Klinger & Cox, 1987; see also Kane et al., 2007, 2017). Second, individuals’ MW rates in the laboratory and daily life correlate positively, thus indicating some stability across contexts (e.g., Antrobus et al., 1967). Third, and most important, there are individual differences in habitual MW with regard to various phenomenological characteristics such as its emotional valence, vividness, and temporal orientation (Singer & Antrobus, 1972). Related to this, the *Imaginal Processes Inventory* (IPI) of Singer and Antrobus (1972), a widely used instrument for measuring daydreaming, lists 28 dimensions measured by a total of 344 items. These dimensions have subsequently been reduced to three higher level factors labelled ‘guilty-dysphoric’, ‘positive-constructive’, and ‘poor-attention’ daydreaming or habitual MW (Giambra, 1977; Huba et al., 1981; Singer & Kolligian, 1987). Accordingly, positive-constructive forms of habitual MW are hypothesized to be beneficial for cognition, creativity, problem solving, and future planning (Singer, 1975). In contrast, poor-attention MW is characterized by an individual’s susceptibility for internal and external distraction, and bears resemblance to what has typically been associated with TUTs in recent research. Consequently, individuals scoring high on this dimension are thought to have difficulties focusing their attention on a current activity, because they are easily distracted by self-generated thoughts or external events.

Empirically, studies referring to these higher-order factors of habitual MW have demonstrated differential relations between positive-constructive, guilty-dysphoric, and poor-attention MW, on the one hand, and measures of well-being (Giambra & Traynor, 1978), personality (Zhiyan & Singer, 1997), and creativity (Li et al., 2022) on the other. Accordingly, positive-constructive MW tends to be associated with desirable outcomes (e.g., creativity, openness to experience) or at least not associated with negative outcomes (i.e., a non-significant relation to well-being), in contrast to the other two factors. Furthermore, in one of the few studies investigating associations between the three dimensions of habitual MW and TUTs while participants carried out a demanding task, Marcusson-Clavertz et al. (2016) showed that TUTs were positively associated with poor inhibition during the Stroop task only for participants scoring low on the positive-constructive dimension. In other words, there was no evidence that task performance of individuals with a predominantly positive-constructive style of habitual MW was affected by TUTs. Taken together, these studies indicate that positive-constructive forms of MW may relate differently to individual difference factors and behavioural outcomes in comparison to guilty-dysphoric and poor-attention MW. In fact, positive-constructive MW might even be beneficial for certain aspects of cognition (McMillan et al., 2013).

Of particular interest with regard to the hypothesis that some types of MW might be useful is a recent study by Soemer et al. (2019), who included measures of both TUTs and habitual MW using a German version of the Daydreaming Frequency Scale (DDFS) from the IPI. Soemer et al. found that their participants (i.e., eighth-grade students) with a high habitual MW frequency experienced more TUTs during reading, and TUTs were associated with poorer comprehension. At the same time, however, habitual MW frequency was overall *positively* correlated with comprehension (*r* = .33), suggesting that some aspect of habitual MW seemed to benefit comprehension and outweigh the negative impact of TUTs during reading. Soemer et al. (2019) speculated that individuals with higher habitual MW propensity potentially engage in positive-constructive MW more frequently, which, in turn, leads to the generation of more elaborative inferences during reading. These inferences would ultimately lead to the construction of a richer situation model and thus better comprehension (Kintsch, 1988). However, because the authors did not include measures of positive-constructive MW and elaborative thinking in their study, this explanation remains to be tested.

## The current study

This study aimed at investigating the relations between habitual MW and TUTs. We set out to show that different dimensions of habitual MW can have opposing effects on individuals’ thoughts during learning. Specifically, we hypothesized that individuals who habitually engage in positive-constructive MW would be more likely to experience thoughts that promote learning (i.e., elaborative thoughts), whereas individuals who habitually engage in poor-attention MW would experience more TUTs during learning, the result being impaired performance. We chose a task that has previously been used in the context of TUTs and learning a number of times and that also provided us with reasonable types of thoughts that may occur and promote the learning goal. Reading comprehension was our task of choice, because successful text comprehension requires the construction of a rich situation model (Kintsch, 1988), which, in turn, requires the reader to focus on external information (i.e., the text), to elaborate this information, and complement it with prior knowledge (Hannon, 2012). Here, we understand elaborations as the addition of information that clarifies the to-be-remembered information (Hannon, 2012). Elaborations may consist of reader-generated analogies, examples, visualizations, re-statements of main ideas, associations with similar concepts, or comparisons between concepts described in the text. Elaborations are thought to promote learning because they make the information provided in the text more retrievable and because they lead to a better integration of this information into prior knowledge.

Another important process for successful reading comprehension is monitoring, a meta-cognitive process by which readers constantly check their progress of comprehension while reading, and regulate reading behaviour accordingly (Follmer & Sperling, 2018). For example, readers may decide to adjust reading speed or to re-read a section whenever they notice they have been engaging in TUTs (Mills et al., 2017). Comprehension monitoring thus likely exerts positive effects because it enables the reader to recognize deficits in understanding and reading strategy use and, as a consequence, stimulates the reader to generate elaborations or inferences that bridge conceptual gaps or switch to strategies that are better suited for reading a text (Follmer & Sperling, 2018).

## Predictions

In accordance with the hypothesis put forward by Soemer et al. (2019), we expected that positive-constructive MW would promote elaborative thinking and comprehension monitoring. The rationale behind this expectation was that individuals who habitually engage in positive-constructive MW should also be more likely to exhibit this tendency during reading, potentially as a reading strategy. Furthermore, because engaging in elaborative inference-building and monitoring reduces the opportunity for TUTs, we expected that positive-constructive MW would be negatively related to TUTs. As regards poor-attention MW, we expected that individuals who habitually engage in this form of MW would transfer this tendency to the current situation and thus be more likely to experience TUTs during reading. As a result, these individuals should tend to show worse comprehension. We also expected poor-attention MW to be negatively related to elaboration and monitoring, because if one is easily distracted, then thoughts referring to monitoring and elaborations should become less likely.

**Fig. 1:**
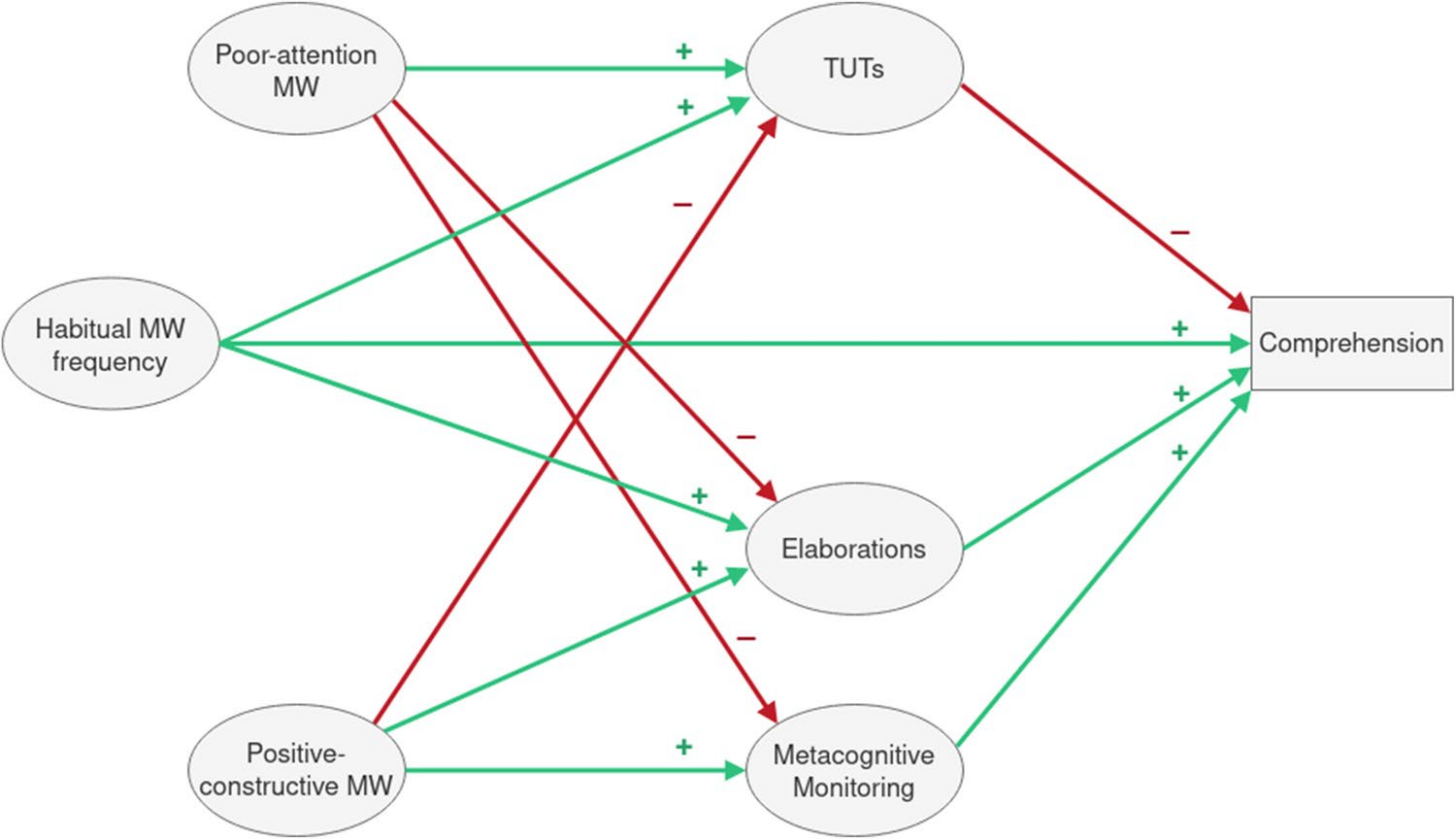
Hypothetical model representing the main research hypotheses. Green arrows (+) indicate a positive association, red arrows (-) a negative association. Covariances between thought contents, covariances between variables of habitual mind wandering (MW), and most direct paths from positive-constructive MW and poor-attention MW to comprehension are not depicted in order to reduce the visual complexity of the figure

Finally, we also included a measure of general habitual MW frequency to have a point of comparison to the study of Soemer et al. (2019). Like that study, we assumed an overall positive relation between habitual MW frequency, on the one hand, and comprehension and TUTs, on the other. In addition, we predicted a positive relation between habitual MW frequency and elaboration. This prediction conforms to the hypothesis of Soemer et al. (2019), who assumed that individuals with higher habitual MW frequency would generate more mental content that promotes comprehension. A visual summary of the most important hypotheses can be found in Fig. 1.

## Materials and methods

We report how we determined our sample size, all data exclusions (if any), all manipulations, and all measures in the study (Simmons et al., 2012).

This study was part of a larger research project investigating various research questions related to MW and a number of other constructs (e.g., reading motivation). Here, we limit our report to those aspects of the study that are directly related to the present research questions (see Soemer et al., 2023). The research project was preregistered on Open Science Framework (OSF; see https://osf.io/j73bm) and approved by the ethics commission of the German Society for Psychology (DGPs).

The study consisted of two sessions separated by about 1 week. The first session assessed the various aspects of habitual MW, the second session assessed reading comprehension and participants’ thought contents during reading (probe-based and questionnaire-based TUTs, elaborative thoughts, comprehension monitoring). The reading session consisted of reading six short expository texts from two subject areas (meteorology and finance), followed by questions pertaining to comprehension and thought contents.

### Participants

The study sample consisted of 200 students from a large university in the east of Germany. 166 students referred to themselves as female, 34 as male. The average age of the participants was 23.25 years (*SD* = 4.50). The sample size was set prior to testing to N = 200 based on our experience with similar studies and studies from other labs. During the course of the study, some participants were excluded for equipment failure (*N* = 1), premature termination of the study (*N* = 6), or because their reading times for two or more texts were two standard deviations away from the average reading time (*N* = 3). These cases were identified prior to data analysis, immediately discarded without further analysis, and replaced with data from new participants to reach the planned sample size of *N* = 200. Participants were rewarded with either 16€ or course credits upon completion of the study.

### Instruments

The instruments for sessions 1 and 2 can be found on the OSF (https://osf.io/hn9pq/).

#### Habitual MW

Our measure of habitual MW was composed of the positive-constructive and the poor-attention scales of Huba et al.’s (1981) *Short Imaginal Processes Inventory* (SIPI) and the *Daydreaming Frequency Scale* (DDFS) from Singer and Antrobus’ *Imaginal Processes Inventory* (IPI; Singer & Antrobus, 1972; German translation taken from Soemer et al., 2019).^1^ Note that the guilty-dysphoric scale of the SIPI was not included in this study because we did not have specific predictions regarding the relation between maladaptive MW and comprehension. Thus, we aimed at capturing three aspects of habitual MW: positive-constructive MW (14 items taken from the SIPI), poor-attention MW (14 items taken from the SIPI), and overall MW frequency (12 items taken from the DDFS). All items were translations from English into German. Some items were linguistically modified to sound more natural compared to the word-by-word translation. There were six response alternatives for each item: ‘*very rarely*‘, ‘*rarely*‘, ‘*rather rarely*‘, ‘*rather often*‘, ‘*often*‘ and ‘*very often*‘ for items asking participants to indicate frequencies, and ‘*strongly disagree*‘, ‘*disagree*‘, ‘*rather disagree*‘, ‘*rather agree*‘, ‘*agree*‘, ‘*strongly agree*‘ for the remaining items.

#### Reading comprehension

Participants read six expository texts, each three about meteorology and finance topics. The texts had a length of about 1,000 words each and were edited to be of moderate difficulty as measured by their readability. Two measures of readability were used, the ‘lesbarhetsindex’ (LIX; Björnsson, 1968) and the Flesch-Reading-Ease formula (FRE; Flesch, 1948). The LIX is computed as the average number of words per sentence relative to the percentage of words that are longer than six letters. Highly readable books, such as children’s books, usually score below 40 on this scale, whereas less readable texts, such as academic papers, usually have values above 60. The FRE is computed by relating the average number of words per sentence to the average number of syllables per word. Here, low values between 0 and 30 are usual scores for academic texts and high values above 80 apply to children’s books. All texts used here scored between 50.35 and 57.46 on the LIX scale, and between 38.19 and 53.37 on the FRE scale, suggesting moderate difficulty for all texts (see Appendix Table 4).

Comprehension was measured after each text by eight questions that required the participants to complete a statement with one out of four highly similar response options (see Appendix 1). Correct choices were scored with one point, incorrect choices with zero points. The number of correct choices served as a measure of comprehension.

#### Thoughts during reading

We used retrospective questionnaires and thought probes to measure participants’ thought contents during reading. The questionnaire items asked participants about the extent to which they remember having engaged in TUTs, elaborative thoughts, and comprehension monitoring during reading. Elaborative thoughts were defined as text-related cognitions that go beyond a text’s content and help to promote comprehension (e.g., mental images, thematic associations), whereas monitoring thoughts were defined as thoughts about one’s ongoing comprehension process (e.g., noticing lacks of comprehension). Each of the three thought categories was assessed by three items (see Appendix 2). There were four response alternatives for each item: ‘*strongly disagree*‘, ‘*rather disagree*‘, ‘*rather agree*‘, ‘*strongly agree*‘.

The retrospective TUT ratings were complemented with thought probes. To this end, participants were interrupted randomly every 60–90 s during reading and asked to indicate whether they had experienced text-related or text-unrelated thoughts prior to the probe. The response options given to the participants were: (1) ‘*I was thinking about something related to the text*‘, (2) ‘*I was thinking about something unrelated to the text*‘, (3) ‘*I was thinking about my reading behaviour*‘, and (4) ‘*I don’t remember*‘. Option (2) was taken as an indicator of MW and divided by the number of interruptions to compute individual MW rates per text.^2^ As a result of this procedure, we obtained on average between 3.55 and 4.05 probes per text, and 1.25 and 8.60 per participant. The TUT rate per participant and text was subsequently calculated as the number of experienced text-unrelated thoughts divided by the number of interruptions.

### Procedure

The study was carried out both in the laboratory and online; specifically, it started out as a laboratory study but was then continued online due to COVID-19 restrictions after half of the planned participants had been tested in the lab. In both cases, however, data were collected over two sessions separated by approximately 1 week and each session took about 60 min. For the laboratory version, participants were seated in a soundproof booth in front of a desktop computer. For online assessment, participants were required to take themselves to a distraction-free environment and use their own computer. Each assessment in both sessions began with instructions that were presented on the computer screen. The experimenter ensured that the participants had fully understood these instructions and helped them in case of any questions about the following task.

At the beginning of the first session, participants were provided with general information about the study and they were informed about the rights and obligations of their participation. Next, they signed a consent form, provided personal information, and then started the assessment of habitual MW. The study software presented the items individually together with a horizontal slider that was to be used to respond to each item on a six-point Likert scale. Participants controlled the slider using the left and right arrow keys and confirmed their selection by pressing the space bar.

The second session served to assess reading comprehension and thought contents during reading (TUT, elaboration, monitoring). The participants were required to read three texts about meteorology and three texts about finance topics (see Appendix Table 4). The presentation order of the texts within each subject area was fixed, whereas the presentation order of the two subject areas themselves was counterbalanced across participants. The participants were instructed to read the texts carefully because their knowledge would be tested afterwards. Moreover, the instructions guided the participants on how to control the text presentation by using the keyboard’s arrow keys. The instructions also referred to the occasional thought probes during reading. and described how to respond by using the number keys on the keyboard. After reading each text, the participants provided the retrospective TUT ratings and then took the comprehension test. At the end of the second session, the experimenter debriefed the participants and authorized the transfer of money or course credits.

### Statistical analysis

The main goal of our analysis was to estimate a structural equation model reflecting our hypotheses regarding the relations between habitual MW, thought contents during reading, and comprehension (see Fig. 1). Before doing so, we first examined a number of important descriptive and distributional properties of all instruments. Next, we carried out confirmatory factor analysis (CFA) to validate the factor structure of the data. In addition, McDonald’s Omega (*ω*) was used as a measure of internal consistency for each scale (McDonald, 1999).

Giving a preview of the results, the literature-driven confirmatory model did not sufficiently fit the questionnaire data even after dropping low-loading items. For this reason, an additional exploratory factor analysis (EFA) was carried out on data taken from another study (Soemer et al., in preparation), and the resulting exploratory model was then confirmed with the current data set. Furthermore, the comprehension measure for one of the texts did not show sufficient internal consistency and was thus removed from the within-subjects analysis. This has to be kept in mind when evaluating the descriptive statistics and the bivariate correlations of the three thought categories, as well as the structural model below.

The analyses were carried out using the *lavaan* package version 0.6-10 (Rosseel, 2012) for *R* version 4.1.2 (https://cran.r-project.org/; last access on March 10, 2022) and M*plus* version 7.4 (Muthén & Muthén, 1998–2015).

## Results

### Descriptive statistics

#### Habitual MW

We calculated means, standard deviations, minimum, maximum, skewness, and kurtosis for each item of the habitual MW frequency (DDFS), as well as the positive-constructive MW and poor-attention MW subscales of the SIPI. All items were properly distributed, most of them having means near the midpoint of the Likert scale and standard deviations within the expected range (see https://osf.io/rs4h3 for detailed results). In particular, there was no indication of ceiling and floor effects or skewed distributions, suggesting that our measurement instruments were overall well calibrated. In addition, internal consistencies of all scales were good, indicating acceptable levels of reliability (DDFS: *ω* = .92; positive-constructive MW: *ω* = .87; poor-attention MW: *ω* = .86).

Correlations^3^ between the items were overall as expected with a few exceptions (see https://osf.io/qdvfc for the full correlation matrix). Most items from the habitual MW frequency scale correlated moderately or strongly with each other, the median correlation being *r* = .52 (.29 < *r* < .80). The items of the positive-constructive and poor-attention MW scales had average correlations of *r* = .32 (.00 < *r* < .74) and *r* = .34 (-.07 < *r* < .66), respectively. The median inter-item correlations between the MW frequency scale and the positive-constructive and poor-attention scales were *r* = .26 (-.08 < *r* < .69) and *r* = .26 (-.25 < *r* < .56), respectively. The median correlation between the positive-constructive and poor-attention scales was small as expected (*r* = .04; -.33 < *r* < .36).

Next, we investigated the factor structure of the three scales. Note that although the DDFS scale was not part of the SIPI, all three scales were analysed together, because they essentially address the same construct and because they are all taken from the larger IPI. In line with the original analysis strategy, we carried out a CFA assuming a three-factor model of habitual MW corresponding to the scales for habitual MW frequency, positive-constructive MW, and poor-attention MW. Because all items were rated on Likert scales with few response options, we treated them as ordinal variables. The fit indices of the resulting CFA model, χ2 = 2253.649 (*p* < .001, *df* = 737), CFI = .810, TLI = .798, RMSEA = .101 (.097; .106), however, did not indicate a satisfactory fit (Kline, 2016). Because removing low-loading items did not solve this issue sufficiently, we decided to change our analysis strategy. We first estimated an exploratory model (EFA) and took the resulting factor structure to subsequently estimate the SEM along the lines of Fig. 1. Because EFA and CFA should not be conducted on the same data set to avoid confirmatory bias, however, we used data from Soemer et al. (in preparation) for the exploratory analysis.^4^

The optimal number of factors for the EFA was determined using Horn’s (1965) parallel analysis. As a result, five factors were suggested, and a corresponding EFA was carried out using ‘geomin‘ rotation. We obtained an acceptable solution, χ2 = 823.114 (*p* < .001, *df* = 590), CFI = .945, TLI = .928, RMSEA = .053 (.044; .061).

Described in words, all but two items of the DDFS loaded on the same single factor (*.63 < λ < .85*). This factor is labelled ‘*Frequency*‘ accordingly. The second factor was comprised of seven items from the positive-constructive scale that all had in common an expression of the value and usefulness of MW for problem solving (*.61 < λ < .82*). This factor is therefore labelled ‘*Value*‘. The third factor contained seven items from the poor-attention scale (*.61 < λ < .80*) and one item from the DDFS (*λ = .60*). Because this factor can be summarized as an individual’s inability to focus on some task, it is labelled ‘*Inattention*‘ accordingly. The fourth factor contained two items from the positive-constructive scale (*λ = .81, .85*) referring to the pleasantness of thoughts while MW, therefore, the label ‘*Pleasant*‘ is used. A fifth factor was obtained with two items from the poor-attention scale referring to external distraction (*λ = .60, .64*). The remaining items did not load sufficiently on any of the factors and were thus discarded. To confirm this factor structure with the current data set, we carried out a subsequent CFA. We obtained a model fit that we deemed acceptable for subsequent analyses, χ2 = 887.921 (*p* < .001, *df* = 367), CFI = .923, TLI = .915, RMSEA = .084 (.077; .091).

#### Thought contents during reading

We analysed the descriptive properties and the factor structure for the three thought contents together, because we considered them to be on the same conceptual level. All indicators seemed to be properly distributed (see https://osf.io/m62dj), and the internal consistencies of the scales were good (TUT: *ω* = .87; elaboration: *ω* = .85; monitoring: *ω* = .84). In addition, correlations between items of the retrospective questionnaire and the probe-based TUT rates taking into account the nested structure of the data were as expected (see https://osf.io/tgaej). Of note, correlations between the retrospective TUT items and the probe-based TUT rates ranged from .58 to .65. Furthermore, the correlation between probe-based TUTs and a latent variable comprised of the three retrospective items was estimated at .71, suggesting broad agreement between the measures.

Next, we modelled the factor structure of the thought contents by means of CFA. For each type of thought content, the corresponding three questionnaire items were loaded on separate latent variables. Probe-based TUT rates were added to the latent variable representing questionnaire-based TUT. Doing so, we obtained an acceptably fitting three-factor solution in accordance with the theoretical thought categories TUT, elaborations, monitoring: χ2 = 238.507 (*p* < .001, *df* = 32), CFI = .974, TLI = .963, RMSEA = .080 (.071; .090). One item of the monitoring scale had a loading of *λ* < .6 and was thus removed from the measurement model, which further improved the fit of the model, χ2 = 117.945 (*p* < .001, *df* = 24), CFI = .988, TLI = .982, RMSEA = .063 (.052; .074), and internal consistency of the monitoring variable (*ω* = .88 instead of .84).

#### Reading comprehension

We computed sum scores per text that would serve as the dependent measure in the structural analysis. There were no issues regarding to the descriptive and distributional properties of the sum scores (see https://osf.io/yh6xj). However, examining the internal consistency, we found that internal consistency was unacceptably low (*ω* = .49) for one of the six texts (‘Clouds‘). For this reason, we decided to remove the complete test from the subsequent analysis. The remaining texts exhibited acceptable levels of internal consistency (*ω* > .60). The comprehension tests correlated lowly or moderately with each other (see https://osf.io/z6q7u).

#### Latent correlations

After establishing the measurement models, we computed latent correlations among all study variables. The latent variables of habitual MW were constructed in accordance with the outcome of the EFA with the exception that the factor referring to external distraction was dropped, as it was technically not concerned with habitual MW. The latent variables of TUTs, elaborative thoughts, and monitoring consisted of each three questionnaire items that were treated as ordinal variables. In addition, probe-based TUT rates were treated as a continuous variable and used as an additional indicator for the latent TUT factor. Comprehension performance for each of the five texts was represented by a sum score and thus modelled as a manifest variable.

Overall, these correlations were in accordance with our hypotheses (Table 1). The frequency factor correlated significantly with the other three factors of habitual MW, and the correlations with the value and inattention factors were quite substantial. In accordance with the theoretical distinction between positive-constructive and poor-attention MW (Huba et al., 1981), the value and pleasant factors correlated strongly with each other, whereas the correlation of these factors with the inattention factor were rather weak.

**Table 1:** Latent bivariate correlations between the measures of habitual mind wandering (MW), thought contents, and comprehension

Variable	(1)	(2)	(3)	(4)	(5)	(6)	(7)
(1) Frequency	-						
(2) Value	.542^***^	-					
(3) Inattention	.718^***^	.124^**^	-				
(4) Pleasant	.191^*^	.568^***^	-.199^*^	-			
(5) TUT	.049	-.108^*^	.147^***^	-.146^**^			
(6) Elaboration	.269^***^	.319^***^	.112^*^	.190^**^	-.458^***^	-	
(7) Monitoring	-.002	.126^**^	-.028	.045	-.713^***^	.385^***^	-
(8) Comprehension	.131^**^	.058	.051	.061	-.468^***^	.223^***^	.322^***^

^*^p < .05. ^**^p < .01. ^***^p < .001

Most importantly, we found significant positive correlations between TUTs during reading and the inattention factor, whereas the correlations with the value and pleasant factors were significantly negative. Interestingly, elaborations were significantly and positively correlated with all four factors of habitual MW. Finally, the value factor was positively correlated with comprehension monitoring.

Comprehension, in turn, was significantly correlated with all three thought variables. Whereas TUTs were negatively associated with comprehension, elaboration and monitoring were positively associated with it. The TUT factor had a strong negative association with monitoring, which should be kept in mind when interpreting the structural model below. As regards the habitual MW scales, the only significant correlation with comprehension concerned the ‘frequency‘ factor, in accordance with Soemer et al. (2019). All other factors of habitual MW were not significantly correlated with comprehension.

### Structural equation modelling

**Fig. 2:**
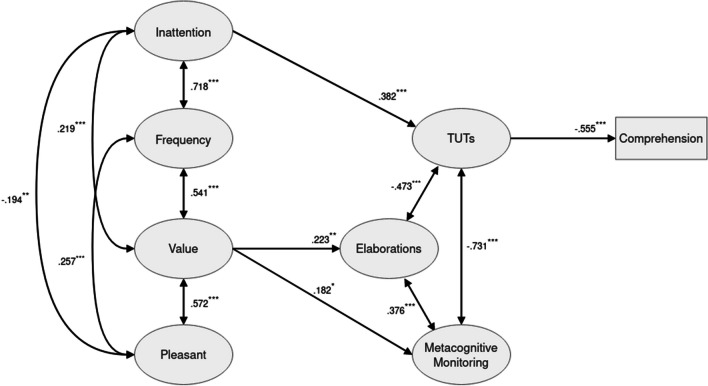
Estimated structural model. Only statistically significant paths are shown (^*^p < .05; ^**^p < .01; ^***^p < .001)

To better understand these associations, we estimated the structural model reflecting our hypotheses with respect to habitual MW and its relation to the mental processes occurring during reading. The latent variables were constructed as described in the previous section. The intercepts in each regression were allowed to vary per participant to account for the fact that there were five texts per participant and, thus, there were each five observations for TUT, elaboration, monitoring and comprehension performance, whereas habitual MW was measured between participants. Model fit was good according to common criteria, χ2 = 1128.335 (*p* < .001, *df* = 602), CFI = .950, TLI = .945, RMSEA = .030 (.027, .032), and the associations between the variables were partially in line with our hypotheses (see Fig. 2 and Tables 2 and 3).

**Table 2:** Standardized coefficients (b), standard errors (SE), expected outcomes (Exp.), p-values, and 95% confidence intervals (CIs) for the directional and bidirectional paths in the model

Directional paths						
Dependent	Predictor	b	SE	Exp.	*p*-Value	95% CI
Comprehension	TUT	-0.555	0.065	b < 0	**<** .001	[-0.682;-0.427]
	Elaboration	-0.042	0.040	b > 0	.297	[-0.120; 0.037]
	Monitoring	-0.039	0.064	b > 0	.546	[-0.165; 0.087]
	Frequency	0.124	0.088	b > 0	.158	[-0.048; 0.295]
	Value	-0.108	0.065	b > 0	.097	[-0.235; 0.020]
	Inattention	0.124	0.085	b < 0	.148	[-0.044; 0.291]
	Pleasant	0.056	0.067	b > 0	.403	[-0.076;-0.188]
TUT	Frequency	-0.167	0.095	b > 0	.079	[-0.354; 0.019]
	Value	-0.134	0.070	b < 0	.056	[-0.271; 0.003]
	Inattention	0.382	0.091	b > 0	**< **.001	[ 0.204; 0.560]
	Pleasant	0.057	0.076	b < 0	.454	[-0.093; 0.207]
Elaboration	Frequency	0.209	0.117	b > 0	.074	[-0.020; 0.438]
	Value	0.223	0.066	b > 0	**< **.01	[ 0.094; 0.353]
	Inattention	-0.085	0.108	b < 0	.432	[-0.297; 0.127]
	Pleasant	-0.004	0.087	b > 0	.961	[-0.174; 0.166]
Monitoring	Frequency	-0.088	0.103	b > 0	.388	[-0.289; 0.113]
	Value	0.182	0.084	b > 0	**< **.05	[ 0.017; 0.347]
	Inattention	-0.009	0.086	b < 0	.915	[-0.179; 0.160]
	Pleasant	-0.019	0.088	b > 0	.832	[-0.192; 0.155]
Bidirectional paths						
Variable 1	Variable 2	b	SE	Exp.	*p*-Value	95% CI
Frequency	Value	0.541	0.046	b > 0	**< **.001	[ 0.450; 0.632]
Frequency	Inattention	0.718	0.037	b > 0	**< **.001	[ 0.645; 0.791]
Frequency	Pleasant	0.257	0.067	b > 0	**<** .001	[ 0.125; 0.389]
Value	Inattention	0.219	0.056	b < 0	**<** .001	[ 0.109; 0.328]
Value	Pleasant	0.572	0.054	b > 0	**< **.001	[ 0.466; 0.679]
Inattention	Pleasant	-0.194	0.070	b < 0	**< **.01	[-0.331;-0.058]
TUT	Elaboration	-0.473	0.033	b < 0	**< **.001	[-0.538;-0.408]
TUT	Monitoring	-0.731	0.025	b < 0	**< **.001	[-0.781;-0.681]
Monitoring	Elaboration	0.376	0.041	b > 0	**< **.001	[ 0.295; 0.456]

**Table 3:** Standardized coefficients (b), standard errors (SE), p-values, and 95% confidence intervals (CIs) for the indirect effects of habitual mind wandering (MW) on comprehension in the model

Predictor	Mediator path	b	SE	p-Value	95% CI
Frequency	TUT	0.093	0.055	.091	[-0.015; 0.200]
	Elaboration	-0.009	0.009	.342	[-0.027; 0.009]
	Monitoring	0.003	0.007	.642	[-0.011; 0.018]
	Total Indirect	0.087	0.054	.102	[-0.017; 0.192]
Value	TUT	0.074	0.040	.066	[-0.005 ;0.153]
	Elaboration	-0.009	0.009	.321	[-0.028; 0.009]
	Monitoring	-0.007	0.012	.559	[-0.031; 0.017]
	Total Indirect	0.058	0.036	.107	[-0.012; 0.128]
Inattention	TUT	-0.212	0.060	**< **.001	[-0.330;-0.094]
	Elaboration	0.004	0.006	.519	[-0.007; 0.014]
	Monitoring	0.000	0.003	.915	[-0.006; 0.007]
	Total Indirect	-0.208	0.057	**<** .001	[-0.319;-0.097]
Pleasant	TUT	-0.032	0.043	.461	[-0.116; 0.053]
	Elaboration	0.000	0.004	.961	[-0.007; 0.007]
	Monitoring	0.001	0.008	.840	[-0.006; 0.008]
	Total Indirect	-0.031	0.040	.440	[-0.109; 0.047]

#### Associations with thought contents

There was no evidence that habitual MW frequency predicted TUTs during reading in our participant sample, as all three thought content variables were not significantly related to this aspect of habitual MW. However, the value factor was positively related to both elaboration and monitoring after controlling for other dimensions of habitual MW, meaning that individuals who found their MW episodes useful tended to generate more elaborations and monitor their thoughts during reading to a greater extent. In line with our expectations, the inattention factor predicted TUTs during reading, the sign of the coefficient being in the expected positive direction. That is, individuals that had indicated a stronger tendency to be distracted by their thoughts in everyday life also reported more TUTs during reading.

#### Associations with comprehension

The small zero-order correlation between the frequency factor and comprehension was reduced and non-significant in the structural model. There was no evidence for any indirect associations of this factor via any of the thought content variables (Table 3). In contrast, there was a significant association between the inattention factor and comprehension mediated by TUTs, as could be expected given the theoretically close relation between one’s habitual inattention in daily life and inattention during specific tasks. This indirect association seemed to account exclusively for the total indirect association between the inattention factor and comprehension (Table 3). There were no other significant direct or indirect associations of any of the factors of habitual MW on comprehension.

## Discussion

The present article set out to investigate the relations between habitual MW, TUTs, and other thought processes that occur while individuals carry out a cognitively demanding learning task. Based on prior research (Huba et al., 1981), we expected to find individual differences in overall habitual MW frequency and two specific forms of habitual MW, positive-constructive and poor-attention MW. Furthermore, we hypothesized that individuals who predominantly engage in the poor-attention type of habitual MW would experience more TUTs, whereas individuals who predominantly engage in positive-constructive type of habitual MW would tend to generate more mental content fostering successful learning (i.e., elaboration and comprehension monitoring).

The results were partially in accordance with our expectations. We found that a five-factor model of habitual MW fit our data better than the theory-driven three-factor model. We obtained a MW frequency factor, two factors of poor attention (inattention and external distraction, the latter being dropped in the structural model), and two factors of positive-constructive MW (value-oriented MW and pleasant MW). We then estimated a model that explored the relations between these factors, on the one hand, and thought contents during reading, on the other.

### Habitual MW frequency

While replicating the results of Soemer et al. (2019) of an overall positive, albeit weak, zero-order correlation between habitual MW frequency and comprehension, we did not find evidence for a negative association of this factor with comprehension mediated by TUT found in that study. In fact, the bivariate correlation between habitual MW frequency and TUT rates was weak and not significant, in contrast to what was reported by Soemer et al. (2019). To explain these different outcomes, one must keep in mind that the two studies differed in their participant samples (university students vs. middle school students), testing procedure (individual vs. group testing), reading material (physics and finance vs. biology) and also the time between the administration of the questionnaires and the reading study (delayed vs. immediate). We assume that particularly age-related differences in executive control capabilities might have caused the different associations with TUT. MW research has repeatedly shown that executive control underlies the suppression of TUTs during reading (e.g., Soemer & Schiefele, 2020). At the same time, executive control follows a developmental trajectory and in particular inhibitory functions may not be fully developed before early adulthood (e.g., Durston et al., 2006). For this reason, the middle school students in Soemer et al.’s study might not have been as capable as our university students to suppress unwanted TUTs, and this might be particularly true for individuals with a strong habit to MW; in other words, university students who frequently engage in MW in daily life might be more successful in suppressing their TUTs during reading compared to middle school students, because their executive control is better developed. With regard to the smaller association of habitual MW frequency with comprehension, this might again be due to age-related or topic-related differences or other factors, and we suggest that future research investigates these factors more thoroughly.

**Fig. 3:**
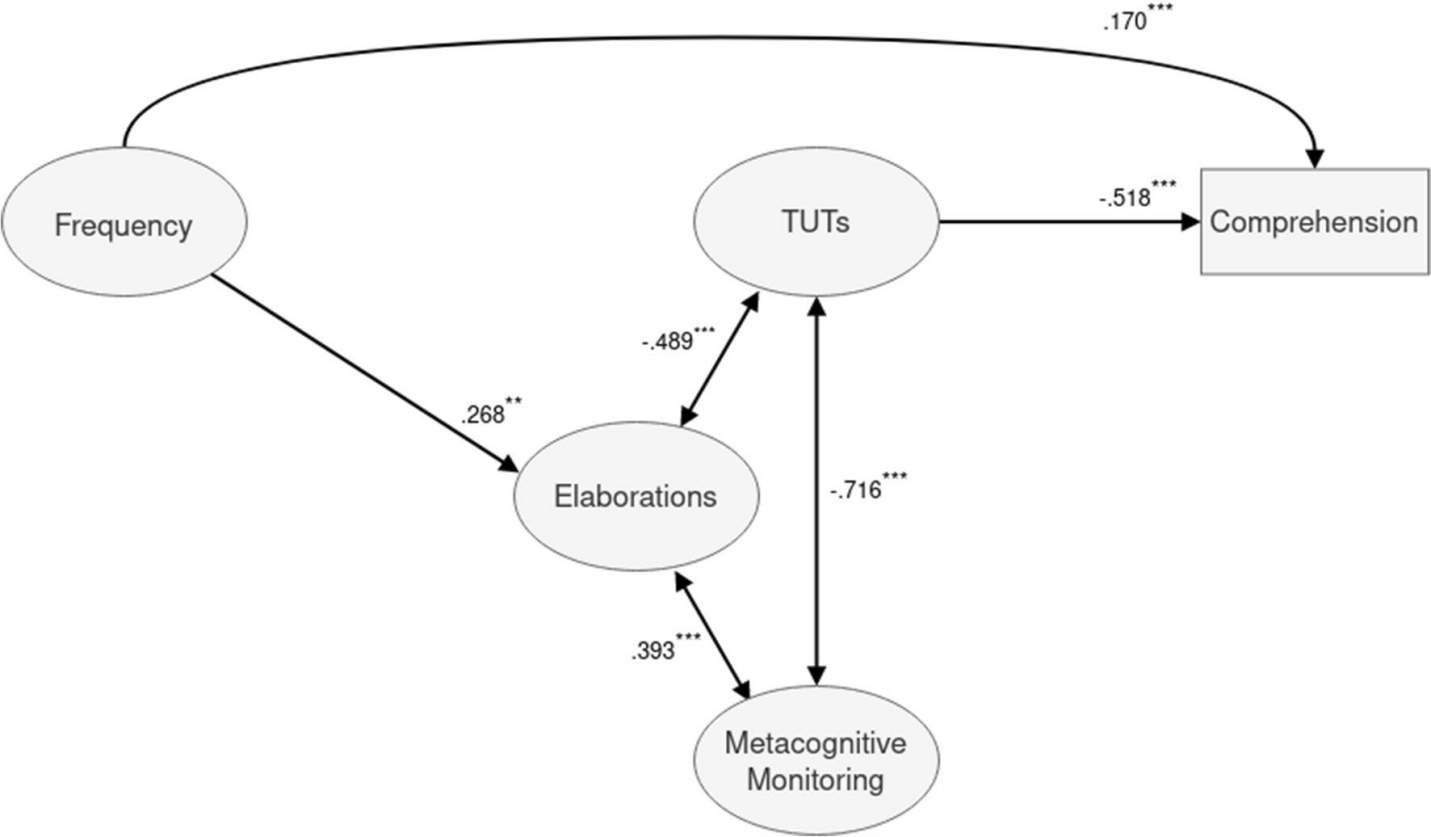
Estimated structural model with habitual mind-wandering (MW) frequency being the only exogenous variable. Only statistically significant paths are shown (^*^p < . 05; ^**^p < .01; ^***^p < .001)

The question remains what mediator processes may underpin the positive relation between habitual MW frequency and comprehension on a bivariate level. According to Soemer et al. (2019), habitual mind-wanderers may overall generate more content that can be useful for completing a task. In the present case, this tendency should manifest itself in a positive relation between habitual MW frequency and the generation of elaborations during reading. Whereas we found evidence that this is the case in terms of a significant bivariate correlation between the two constructs, however, this association was weak and not significant in the path model. One may suspect that the substantial correlations of frequency with the other factors of habitual MW, in particular, the correlation between the frequency and the inattention factor (*r* = .72) could in part account for this result. Indeed, estimating a simple model with frequency being the only factor of habitual MW, and TUT, monitoring and elaboration being the mediators (see Fig. 3), χ2 = 295.995 (*p* < .001, *df* = 161), CFI = .985, TLI = .982, RMSEA = .029 (.024, .034), we found a significant association between MW frequency and elaboration (*β* = .027; *p* < .001). However, there was again no evidence for a mediation of the association between MW frequency and comprehension by elaboration (*β* = -.014; *p* = .165) even in this simple model, whereas the direct association between MW frequency and comprehension was significant (*β* = .170; *p* < .001). This suggests that other currently unknown processes may contribute to this relation, and this issue should be further investigated in future studies.

### Positive-constructive MW

Individuals who scored high on the value factor, generated more elaborations, more frequently monitored their reading behaviour, and experienced fewer TUTs. The associations of this factor with elaboration and monitoring also remained in the path model, whereas the negative association with TUTs became non-significant. The pleasant factor was not significantly related to any of the thought content variables in the structural model despite having a positive significant zero-order correlation with elaboration and a negative significant zero-order correlation with TUTs. Given that this factor showed a substantial correlation with the value factor, however, it seems that it may not explain sufficient unique variance in elaboration.

Overall, our results support the initial hypothesis stating that individual tendencies to engage in positive-constructive MW may promote processes that potentially support reading. On the other hand, one might object that these beneficial processes themselves did not always significantly affect comprehension – in particular, the paths from elaboration to comprehension were not significant – and, thus, engaging in positive-constructive MW might not help comprehension in reality.

**Fig. 4:**
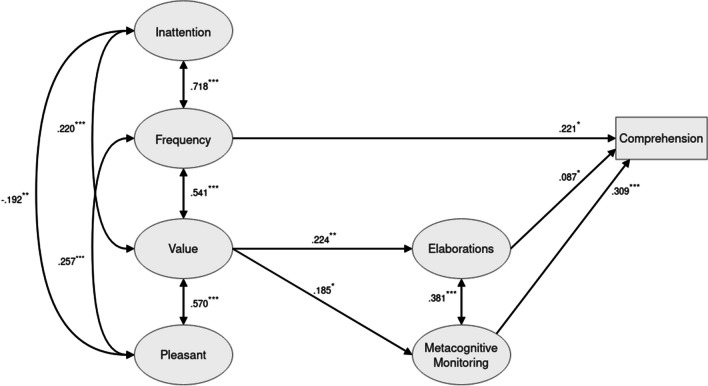
Estimated structural model with task-unrelated task (TUT) being removed as mediator process. Only statistically significant paths are shown (^*^p < . 05; ^**^p < .01; ^***^p < .001)

While this could be the case, we would like to point out that both elaboration and monitoring were substantially correlated with TUT rates, suggesting that all three predictors explained partially overlapping variance in comprehension. And of all three variables, TUT seemed to be the strongest, thus reducing the effects of the other two on comprehension. Indeed, removing TUT from the model (Fig. 4), χ2 = 989.280 (*p* < .001, *df* = 475), CFI = .936, TLI = .929, RMSEA = .033 (.030, .036), led to a significant association of monitoring with comprehension (*β* = .309; *p* < .001) and of elaboration with comprehension (*β* = .087; *p* < .05). In addition, the ‘frequency‘ factor was directly and significantly related to comprehension (*β* = .221; *p* < .05), and the ‘value‘ factor was significantly related to comprehension mediated by comprehension monitoring (*β* = .057; *p* < .05). Thus, we suggest that methodological improvements, in particular, a fine-tuning of the measurements for the process variables might give us a clearer picture in the future.

### Poor-attention MW

Individuals who predominantly engaged in the inattentive type of habitual MW experienced more TUTs during reading, and this seemed to be an important reason for their lower comprehension scores. In addition, TUT rates seemed to account for the complete indirect association of MW inattention with comprehension. This outcome makes sense given that the questionnaire items from the MW inattention factor largely describe a situation in which an individual fails to focus on a given task because of distracting thoughts. Thus, the MW inattention factor provides a good prediction of how frequent individuals may experience TUTs in a specific situation. In contrast to our expectations, however, there was no evidence that inattention ratings (negatively) predicted the occurrence of elaboration and monitoring thoughts during reading.

## Conclusion

Taken together, the results suggest that individuals with a propensity to engage in positive-constructive forms of habitual MW generate more beneficial thought contents during reading. Conversely, individuals with a propensity to engage in the poor-attention type of habitual MW are more likely to be distracted from reading which can impair information intake and, thus, building a sufficient text base and situation model.

The results highlight different sides of the phenomena commonly termed MW and their effects on cognition and learning. Whereas most research in the last few decades has been specifically focusing on the ‘poor-attention’ aspect of habitual MW and, more specifically, the occurrence of TUTs, we emphasize that MW in the broad sense (i.e., as spontaneous or ‘freely-moving’ thought; Christoff et al., 2016) can have both positive and negative associations with the very same task depending one’s style of MW. In particular, while replicating the negative association between poor-attention MW and TUTs, we showed for the first time that positive-constructive habitual MW can have positive associations with thoughts that potentially promote reading, specifically, elaborations and comprehension monitoring. These results partially (though not completely) support a hypothesis put forward by Soemer et al. (2019) who speculated that individuals with strong propensity to mind-wander in daily life might also generate more mental content while reading.

It is also worthwhile to compare the present results to prior studies investigating the effects of different forms of habitual MW. In the study by Marcusson-Clavertz et al. (2016), Stroop task performance was negatively affected by TUTs only for those participants who scored low on the positive-constructive scale of the SIPI suggesting that individuals with a predominantly positive-constructive style of habitual MW may not be affected by TUTs. Going beyond this result, we here provided evidence suggesting that the individual tendency to engage in certain forms of positive-constructive MW may even promote task-related thinking.

In an attempt to investigate the validity of the probing method for measuring TUTs, Kane et al. (2021) compared whether TUT reports vary depending on the specific way participants are probed during attentionally demanding tasks. Kane et al. (2021) also analysed the relationship between TUT rates in the different probe conditions and several questionnaire measures, including a measure of positive-constructive MW that was constructed by grouping together items from various IPI dimensions (Singer & Antrobus, 1972). As a result, most correlations between this measure and TUT rates were not significant, except for when participants were asked to indicate the intentionality of their TUTs as well. In that case, participants who scored higher on Kane et al.’s positive-constructive scale reported higher TUT rates, and additional analyses showed that this primarily concerned intentional TUT rates (*r* = .22[.02, .40] as compared to unintentional TUTs (*r* = .18 [−.02, .36]). One apparent difference between their and our study, however, is that positive-constructive MW was unlikely to benefit performance in their attentionally demanding laboratory tasks in the first place, because elaborating on the task or thinking about how well one performs on the task likely would not help performance. Even worse, any thought directed to something other than the specific task or external stimuli likely impairs performance in such tasks.

However, it would be interesting to explore in the future the link between the intentionality of MW during reading and the different dimensions of the SIPI (in particular positive-constructive and poor-attention MW). It is possible that mental processes associated with positive-constructive MW are generally more deliberate, and, in the case of reading, individuals might constantly reassess whether engaging in elaboration or monitoring will benefit comprehension, which might be one reason for why we observed a negative association between the individual tendency to engage in certain forms of positive-constructive MW and TUTs. Taken together, future research will have to determine in more detail the boundary conditions under which the spontaneous thoughts may benefit learning, and what other forms of MW may underlie the observed positive association between habitual MW frequency and comprehension.

## Data Availability

The data that support the findings of this study are openly available in OSG at https://osf.io/jpfa9/.

## Appendix 1

### Example comprehension questions

The following questions are approximate translations of the original German comprehension tests.

Example questions for the ‘meteorology‘ texts:

1. *If the difference in solar radiation were the only factor in the generation of wind, then the wind on the ground*… (correct answer: a)
  - … *would always blow from the north*
  - … *would always blow from the south*
  - … *and in the higher layers of air would always blow toward the pole*
  - … *and in the higher layers of air would always blow toward the equator*
2. *On coasts ,*… (correct answer: c)
  - … *ascending winds usually form over the sea during the day*
  - … *low-pressure areas usually form on the ground over the sea during the day*
  - … *low-pressure areas usually form on the ground over the land mass during the day*
  - … *sinking winds usually form over the land mass during the day*

Example questions for the ‘finance‘ texts:

1. *An essential precondition for a speculative bubble is a* …‘ (correct answer: b)
  - … *stable price for an economic good over an extended period of time*
  - … *rising price for an economic good over an extended period of time*
  - … *rising price for an economic good over a short period of time*
  - … *stable price for an economic good over a short period of time*
2. *A key innovation of the first stock corporations was that* … (correct answer: a)
  - … *shareholders could not reclaim their invested capital*
  - … *shareholders could no longer get rid of their shares in the company*
  - … *company's assets were determined by the value of the company's shares*
  - … *company's assets grew steadily as shares were distributed*

## Appendix 2

### Retrospective questionnaire for the assessment of thought contents during reading

The following items are approximate translations of the original German questionnaire.

Instruction: ‘*Now, please rate your level of agreement with some statements about your thoughts while reading*.‘

Task-unrelated thoughts:

*I often thought about something unrelated to the text.*

*I was able to concentrate on the text with ease.* (reversed coding)

*I have read the text carefully.* (reversed coding)

Elaboration:

*The contents addressed in the text stimulated my imagination.*

*I constantly had interesting associations while I was reading.*

*I visualized the contents addressed in the text.*

Monitoring:

*I made sure that I sufficiently understood the concepts addressed in the text.*

*I made myself aware that my thoughts should be focused on the text.*

*I repeatedly ensured that I actually understood the issues addressed in the text.*

## Appendix 3

**Table 4:** Readability calculations

Title	Subject	Word count	Word length	Sentence count	Sentence length	Number of syllables	LIX	FRE
Speculation Bubbles	Finance	1088	5.60	51	21.33	1.80	53.22	53.37
Paper Money	Finance	956	6.05	51	19.51	1.91	55.18	48.76
Central Banks	Finance	1029	6.30	51	21.89	2.05	57.46	38.19
Winds	Weather	1060	5.63	51	21.20	1.85	50.35	50.58
Clouds	Weather	1169	5.99	57	20.51	1.98	57.12	43.66
Atmospheric Pressure	Weather	971	6.09	46	21.11	1.96	56.85	44.23

## Footnotes

1 It must be acknowledged that the SIPI items were translated into German by the first author without examining potential changes in meaning caused by the translation. To address this potential issue retrospectively, we asked a native speaker of English who was blind to the purpose of this study to translate the German items back into English. The comparison between original and back-translated items can be found on the OSF (https://osf.io/qke3h).

2 One could argue that option (4) might as well indicate that participants had been engaging in MW prior to the interruption. However, this option was rarely chosen (4% of all thought samples) and, in fact, it did not matter whether or not we included this option in our calculation of MW rates.

3 Polychoric correlations were preferred over Pearson correlations due to the small number of response categories. They were computed using the ‘polycor’ package version 0.8-1 in R.

4 Also in this data set, all scales were reliable in terms of their internal consistency (DDFS: *ω* = .93; positive-constructive MW: *ω* = .84; poor-attention MW: *ω* = .87).
